# *Rosa canina* Extracts Have Antiproliferative and Antioxidant Effects on Caco-2 Human Colon Cancer

**DOI:** 10.1371/journal.pone.0159136

**Published:** 2016-07-28

**Authors:** Sandra Jiménez, Sonia Gascón, Asunción Luquin, Mariano Laguna, Carmen Ancin-Azpilicueta, María Jesús Rodríguez-Yoldi

**Affiliations:** 1 Applied Chemistry Department, Public University of Navarre, Pamplona, Spain; 2 Department of Pharmacology and Physiology, CIBERobn, IA2, IIS Aragón, Veterinary Faculty, University of Zaragoza, Zaragoza, Spain; 3 Institute of Chemical Syntheses and Homogeneous Catalysis, CSIC, Inorganic Chemistry Department, University of Zaragoza, Zaragoza, Spain; University of Sassari, ITALY

## Abstract

The *in vitro* antiproliferative and antioxidant effects of different fractions of *Rosa canina* hips on human colon cancer cell lines (Caco-2) was studied. The compounds tested were total extract (fraction 1), vitamin C (fraction 2), neutral polyphenols (fraction 3) and acidic polyphenols (fraction 4). All the extracts showed high cytotoxicity after 72 h, both low and high concentrations. The flow cytometric analysis revealed that all the fractions produce disturbances in the cell cycle resulting in a concomitant cell death by an apoptotic pathway. Changes in the redox status of Caco-2 cells in response to *Rosa canina* hips were determined. Cells were exposed to hydrogen peroxide in presence of plant fractions and the production of Reactive Oxygen Species (ROS) was significantly decreased. Therefore, our data demonstrate that rosehip extracts are a powerful antioxidant that produces an antiproliferative effect in Caco-2 cells. Therefore, these results predict a promising future for *Rosa canina* as a therapeutic agent. Thus, this natural plant could be an effective component of functional foods addressed towards colorectal carcinoma.

## Introduction

For a long time it has been believed that implementing diet with antioxidants diminish risk of suffering different illnesses, although this belief is now under revision [1,2]. Antioxidants role, in fruits and vegetables, is to avoid biological oxidation involved in cellular damage and these kind of molecules are also used in the food industry to avoid oxidation worsening [3]. García-Alonso *et al*. [4] reported that dietary constituents can exert significant modulatory effects on cell proliferation, cytotoxicity and oxidative reactions in cellular systems. Numerous *in vitro* studies indicate that plant secondary metabolites can potentially affect diverse processes in mammalian cells, including gene expression, apoptosis, low-density lipoprotein oxidation, intracellular signaling, P-glycoprotein activation and the modulation of enzyme activities associated with carcinogen activation and detoxification [5–7]. Phenolic compounds, primary antioxidants, present antiproliferative properties and inhibit or delay oxidation by scavenging free radicals. Secondary antioxidants work through several mechanisms such as binding of metals ions, scavenging ROS, absorbing UV radiation, converting hydroperoxides to non-radical species or deactivating singlet oxygen [8,9]. Reactive oxygen species are produced as a consequence of the cellular metabolism and their concentration can be increased due to ambience pollution, tobacco consumption, etc. An excess of these reactive compounds can produce damage in some molecules of the animal cells such as lipids, proteins and DNA [10]. In humans, ROS can produce diseases like inflammatory disorders and cancer [11], because of the cell damage that they provoke. At low and moderate concentrations, ROS and Reactive Nitrogen Species (RNS) have beneficial effects and involve physiological roles in cellular responses, as for example as a defense against infectious agents, in the function of a number of cellular signaling pathways, and the induction of a mitogenic response. It is known that various ROS-mediated actions in fact protect cells against ROS-induced oxidative stress and re-establish or maintain “redox balance” [12–14]. Redox and antioxidant systems are among the most promising targets for functional food science [15,16]. Given the difficulty of synthesizing molecules with antioxidant ability, the extraction of these compounds from natural sources such as vegetables or fruits is very important [8]. The *Rosa* gender contains about 100 species that are widely distributed in Europe, Asia, North America and the Middle East. Rosehips, the pseudo-fruits of the roses (*Rosa* spp), have been used in many cultures like countries of Central Europe as food and with pharmaceutical purposes and have attracted more attention in recent years because of their supposed and documented health properties [17]. Consumption of wild fruits of *Rosa Canina* in traditional herbal teas, marmalades, jellies, jams, yoghurts, soups [18], food supplements [19], etc. is common in countries of Central Europe (Germany, Hungary, Austria, Serbia, Poland …) for the treatment of cold and influenza. In general, rosehips present high concentrations of ascorbic acid, phenolic compounds and healthy fatty acids [20]. However, the content of these substances can change depending on different factors such as the geographical origin of the rosehip or the variety of rose from which the rosehips come [21].

On the other hand, cancer is a major cause of death around the world. One of the characteristics attributed to antioxidants is that they decrease the risk of suffering cancer. Gallaher and Trudo [22] among other authors have suggested that high consumption of fruit and vegetables decreases the risk of colon cancer. Polyphenols could play an important role in anticancer and their effects have been observed on different organs. Many polyphenols, such as proanthocyanidins, flavonoid, resveratrol, tannins, epigallocatechin-3-gallate, gallic acid and anthocyanin, have been tested; all of them showed protective effects in some models although their action mechanism were found to be different [23]. Wenzel *et al*. [24] showed the putative anticancer activity of 2-phenyl-4H-1-benzopyran-4-one, the core structure of the flavones, on HT-29 human colon cancer cells. Flavone was found to reduce cell proliferation in HT-29 cells and to potently induce differentiation as well as apoptosis. In this way, many polyphenols have demonstrated chemo protection, such as antiproliferation, antioxidation, estrogenic/antiestrogenic activity, induction of cell cycle arrest or apoptosis, induction of detoxification enzymes, regulation of the host immune system and changes in cellular signaling [25]. In addition, quercetin exert a preferential cytotoxic effect on dividing colon carcinoma HT-29 and Caco-2 cells [26]. Extensive research has been carried out on the potential anticancer properties of ellagic acid, mediated by the regulation of cell cycle progression and cell death (apoptosis) in cancer cells [27]. It is believed that phenolic compounds can exert their effects on different signaling pathways such as Mitogen-Activated Protein Kinases (MAPK), Activator Protein-1 (AP-1) or Nuclear Factor–kB (NF-kB) either separately or sequentially, as well as possibly interacting among these pathways, which can offer a complementary and overlapping mechanism of action [28]. It is posited that the additive and synergistic effects of phytochemicals in fruits and vegetables are responsible for their potent antioxidative and anticancer activities and that the benefits of this diet are attributed to the complex mixture of phytochemicals present in whole foods [29].

Therefore, given the importance of having better knowledge on the natural antioxidants action mechanism in carcinogenic cells, the aim of this study is to evaluate the biological properties of different concentrations of *Rosa canina* hips extracts on colon cancer cell line, Caco-2. We evaluate the antioxidant activity of vitamin C, neutral and acidic polyphenols from *Rosa canina* hips and its possible role in protecting against DNA from oxidative damage. The effect of the rosehip extracts on the inhibition of proliferation of the colon cancer cell line, Caco-2, and on cellular cycle and apoptosis are also investigated.

## Materials and Methods

### Plant materials, HPLC antioxidant analysis and antioxidant activity

The study has been carried out with rosehips, 2014 harvest, from *Rosa canina* specie. The rosehips used were collected in Mariola Mountain in the field called “Ca’l retor” which is located in Agres (Alicante) municipality, with the permission of its owner D. Fidel Pascual Molins. The firm that provided the rosehips samples is Herbes del Molí (Avenida Constitución, 5, 03827 Benimarfull, Alicante, Spain) and has the necessary permissions for this type of activity. On the other hand, samples does not belong to an endangered or protected species. In this geographical zone, winters are cold with temperatures as low as -15°C while summers are hot with temperatures that can exceed 35°C and even 40°C. Precipitations are very irregular, and can range from 350 to 900 mm/year. Freshly collected rosehips were frozen (-40°C) before undergoing analysis.

#### Preparation, purification, fractioning and HPLC analysis of rosehips

To prepare, purify and fractionate the rosehip extract, the Tumbas *et al*. [19] modified method was used. Rosehips peel and pulp (10 g) were homogenized with ultra-turrax and then macerated for 24 h in a 250 mL acetone:water (80:20 v/v) solution. After this phase, the sample was lyophilized (Telstar, model Cryodos-50, Spain) to eliminate solvents and all the antioxidants are present in the residue obtained (total extract, Fr 1). This extract (Fr 1) was then fractionated using a solid phase chromatography to obtain three different antioxidants fractions. First of all, the residue was solved in sulphuric acid (5 mL, 0.5 M), the solution was centrifuged (15000 rpm, 30 min) and passed through a Chromabond C-18 cartridge (Scharlab S.L., Barcelona) previously conditioned (2 mL of methanol and 5 mL of 5 mM H_2_SO_4_). Polar substances, such as vitamin C, were extracted with 2 mL of 5 mM H_2_SO_4_ (Fr 2).

Phenolic compounds retained in the cartridge, were eluted with methanol (2 mL) and distilled water (5 mL). From this solution, considered as purified rosehip extract, neutral and acidic phenols were extracted and fractioned. In brief, the purified rosehip extract pH was adjusted to 7.0 (using a 2 M NaOH solution) and passed through a preconditioned Chromabod cartridge with 8 mL of methanol followed by 4 mL of distilled-deionized water. The column was washed with 10 mL of distilled-deionized water and neutral phenols (Fr 3) were eluted using methanol (12 mL). The effluent portion pH was adjusted to 2.0 (using 2.0 M HCl) and then it was passed through a cartridge preconditioned with methanol (8 mL) and HCl (4 mL, 0.01 M). Subsequently column was washed with HCl (5 mL, 0.01 M) and the retained fraction (acidic phenols, Fr 4) was eluted using methanol (12 mL). All the fractions (1–4) were dried using the lyophilizer and then were frozen at -40°C until the HPLC analysis.

HPLC analysis was performed with a high-pressure liquid chromatograph (Waters Chromatography Div., Milford, MA) equipped with two 515 pumps, a U6K injector, and a 996 Photodiode Array Detector used at different wavelengths for individual compounds (from 200 to 600 nm). The column used was an Atlantis dC 18 reverse phase (150 mm × 4.6 mm i.d., 5 μm particle size) and for chromatographic control, Empower 2.0 software was employed. Two mobile phases A (0.1% phosphoric acid) and B (acetonitrile), both from Sharlab, were used for neutral and acidic phenolic compounds analysis. The flow rate was 1 mL/min with the linear gradient profile: 0–20 min, from 10 to 22% B; 21–40 min, from 22 to 40% B; 41–50 min, from 40 to 55% B; 51–60 min, from 55 to 10% B; 61–65 min, equilibration at 10% B. The injection volume of samples, dissolved in methanol, was 10 μL.

**Fig 1:**
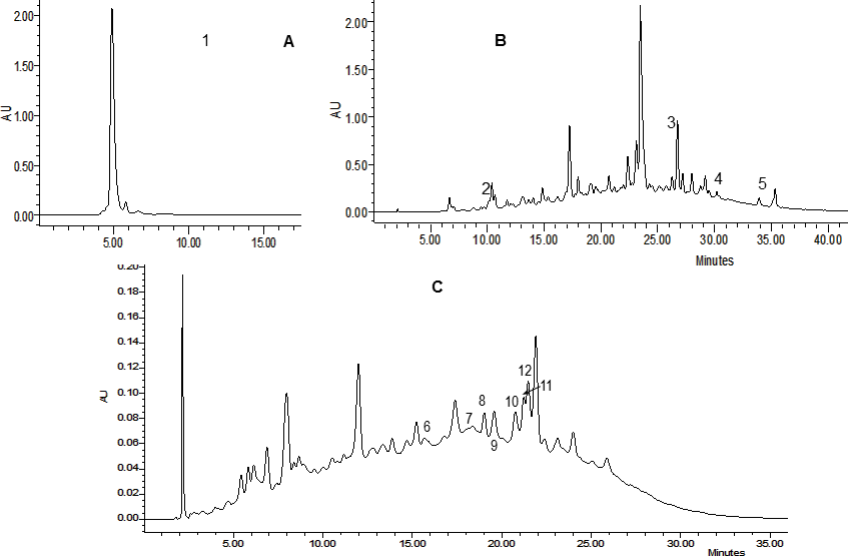
**Example of Fr 2 (A); Fr 3 (B) and Fr 4 (C) chromatograms.** Peaks: (1) ascorbic acid; (2) catechin; (3) rutin; (4) quercetin; (5) myricetin; (6) caffeic acid; (7) coumaric acid; (8) syringic acid; (9) gallic acid; (10) vanillic acid; (11) protocatechuic acid; (12) ellagic acid.

For HPLC analysis of the ascorbic acid, ammonium acetate 0.1 mol/L at pH 5.1 was used as mobile phase. The flow rate was 0.4 mL/min. The samples were dissolved in a solution of metaphosphoric acid (3% w/w) in 8% acetic acid and the injection volume of samples was 10 μL. Fig 1 shows an example of chromatograms of fractions 2, 3 and 4.

#### Antioxidant activity of rosehip extract fractions

Total antioxidant activity was measured according to ABTS method described by Cano *et al*. [30] For this assay, ABTS radical, 2,2’-azino-bis-(3-ethylbenzoithiazolone-6-sulphonic acid), and potassium persulfate were prepared. Radical is formed after 16 h in the dark. To obtain the calibration curve, the absorbance as A_0_ (not inhibited signal) was measured in the UV spectra of 1500 μL of ABTS at 744 nm a Jasco Spectrophotometer V-630 (Washington, USA), then 25 μL of ascorbic acid standard solution was added. After stirring for one minute, the absorbance A (inhibited signal) was registered. This procedure was repeated with six ascorbic acid solutions at different concentrations (90–300 μM) prepared from an initial 4 mM standard solution. The percentage of inhibition is calculated by the equation: % Inhibition = 1-(A/A_0_) x 100. After obtaining the calibration curve, the same procedure, adding 25 μL of *Rose* sample to 1500 μL of ABTS, was used. The total antioxidant activity was expressed as mg of ascorbic acid per gram of dried fruit.

All the calculations and concentrations were referred to dry plant material, this percentage also helps to know the freshness of the fruit. To establish dry plant material percentage, 5 g of rosehips peel and pulp were weighted and kept at 105°C in a heater for 24 h, then they remained in a desiccator for 30 minutes and once cooled they were weighted. The procedure was repeated till constant weight. The calculated dry plant material percentage was 62%. In all cases, n = 4 (two samples of each fraction and two analysis of each sample).

### Biological assays

#### Cell culture

The Caco-2 cells were kindly provided by Dr. Edith Brot-Laroche (Université Pierre et Marie Curie-Paris 6, UMR S 872, Les Cordeliers, France). Two cell clones were selected for further analysis: Caco-2/PD7 (from early passage and heterogeneous) and Caco-2/TC7 (from late passage and homogeneous). These clones exhibited a well-organized brush border at late confluence and no differences in the levels of dipeptidylpeptidase IV activity. Both clones were selected on the basis of differences in the levels of expression of the enzyme sucrose-isomaltase in the brush border of the small intestine and rates of glucose consumption. Expression of sucrose-isomaltase was shown to increase from early to late passages of Caco-2 cells, concomitant with a decrease in the rates of glucose consumption [31]. Caco-2 cells were maintained in a humidified atmosphere of 5% CO_2_ at 37°C. Cells (passages 50–80) were grown in Dulbecco’s Modified Eagles medium (Gibco Invitrogen, Paisley, UK) supplemented with 20% fetal bovine serum, 1% non-essential amino acids, 1% penicillin (1000 U/mL), 1% streptomycin (1000 μg/mL) and 1% amphotericin (250 U/mL). The cells were passaged enzymatically with 0.25% trypsin-1 mM EDTA and sub-cultured on 25 or 75 cm^2^ plastic flasks at a density of 2x10^4^ cells/cm^2^. Culture medium was replaced every 2 days. Cell confluence (80%) was confirmed by microscopic observance. Experiments were performed 24 hours post-seeding to prevent cell differentiation [32].

#### Cell treatment and determination of cytotoxicity

Stock of the *Rosa canin*a fractions were diluted in complete medium to the required concentration. For cytotoxicity screening experiments, cells were seeded in 96-well plates at a density of 4x10^3^ cells/well. The culture medium was replaced with fresh medium (without fetal bovine serum) containing plant fractions 1–4 at concentrations varying from 0 to 1000 mg/L, with an exposure time of 72 h. Thereafter, the cell growth were measured using the sulforhodamine B assay [33]. Cells were fixed with 500 g/L trichlroacetic acid (1 h, 4°C), washed with distilled water and stained with 4 g/L of sulforhodamine B (20 min, room temperature). The plates were then washed with 10 mL/L acetic acid to remove unbound dye. Protein-bound dye were extracted with 10 mmol/L Tris base. Finally, the results were obtained by measuring absorbance with a scanning multiwell spectrophotometer (Biotex Sinergy ht Siafrtd, Vermont, USA) at wavelength of 540/620 nm. The effect on cell growth was expressed as a percentage of the control and calculated as % control. Experiments were conducted in quadruplicate wells and repeated at least three times.

#### Measurements of apoptosis

Human Caco-2 cell line PD7 and TC7 clones were exposed for 72 h with 125 or 1000 mg/L of the 1–4 plant fractions, collected and stained with Annexin V-FTIC according to manufacturer’s recommendation. A negative control was prepared by unreacted cells, that was used to define the basal level of apoptotic and necrotic or dead cells. After incubation, cells were transferred to flow-cytometry tubes and washed twice with temperate phosphate-buffered saline and resuspended in 100 μL Annexin V binding buffer (10 mM Hepes/NaOH, pH 7.4, 140 mM NaCl, 2.5 mM CaCl_2_), 5 μL of the Annexin V-FITC and 5 μL of propidium iodide (PI) to each 100 μL of cell suspension. After incubation for 15 min at room temperature in the dark, 400 μL of 1 X Annexin binding buffer were added and analysed by flow cytometry within 1 h. The signal intensity was measured using a BD FACSAria (New Jersey, USA) and analysed using BD FASCDiva.

#### Propidium iodide staining of DNA content and cell cycle analysis

Human Caco-2 cell lines PD7 and TC7 clones were exposed for 72 h with 125 or 1000 mg/L of the 1–4 plant fractions. Cells were fixed in 70% ice-cold ethanol and stored at 4°C for 24 h. After centrifugation, cells were rehydrated in phosphate-buffered saline and stained in PI (50 μg/mL) solution containing RNase A (100 μg/mL). PI stained cells were analysed for DNA content in a BD FACS Array (New Jersey, USA) equipped with an argon ion laser. The red fluorescence emitted by PI was collected by 620 nm longer pass filter, as a measure of the amount of DNA-bound PI and displayed on a linear scale. Cell cycle distribution was determined on a linear scale. The percentage of cells in cycle phases was determined using MODIFIT 3.0 verity software.

#### Intracellular peroxides (ROS) formation

The cells were seeded in 96-well plates at a density of 4x10^3^ cells/well. The production of ROS was assessed using the dichlorofluorescein assay [34]. Caco-2 cells were cultured for 24 h before oxidative stress induction, and then incubated with 100 μL of serum-free culture media with two concentrations of the 1–4 fractions (125 or 1000 mg/mL) in 96 multiwell plates for 24 h. Then medium was removed, cells were washed twice with phosphate buffered saline and incubated for 1h with 100 μL of 20 μM 2’,7’-dichlorofluorescein diacetate (DCFH-DA) in PBS at 37°C. After this period cells were washed and re-suspended in serum-free culture media (control) or PBS supplemented with 20 mM H_2_O_2_. The formation of the fluorescence oxidized derivative of DCF-DA was monitored at emission wavelength of 535 nm and excitation wavelength of 485 nm in a multiplate reader. A measure at time “zero” was performed, cells were then incubated at 37°C in the multiplate reader, and generation of fluorescence was measured after 20 minutes. ROS production is expressed as fluorescence arbitrary units, expressed as a percentage from cells fluorescence with H_2_O_2_ without preincubation in fraction plants.

### Statistical analysis

All results are expressed as means ± SEM. Means were compared using a one-way analysis of variance (ANOVA). Significant differences at p < 0.05 were compared using a Bonferroni’s Multiple Comparison Test. The statistical analysis and the graphics were performed using the GraphPad Prism Version 5.02 program on a PC computer.

## Results and Discussion

### Antioxidants in each rosehip extract fractions

In *Rosa canina* hips extracts, the amount of vitamin C found was 101±1 mg/Kg of dry fruit (Table 1 and S1 Table). Vitamin C concentration, in this sample, was six times higher than that found by Tumbas *et al*. [19] in rosehips of *Rosa canina* from Serbia. However, it was lower than those found by Demir *et al*. [18] in *Rosa canina*, *Rosa dumalis*, *Rosa gallica*, from Turkey and from those found by Türkben *et al*. [35] in *Rosa canina* also from Turkey. Strålsjö *et al*. [36] found that the different ascorbic acid content in the rosehips is due to many factors but mainly to the fact that dried rosehips present less ascorbic acid content than fresh rosehips. The total antioxidant capacity of this fraction was 38.5 mg of vitamin C/g of dry sample. Total neutral polyphenols components (myricetin, rutin, catechin and quercetin) in *Rosa canina*, was 40.8 mg/kg of dry fruit. Among neutral polyphenols found in these rosehips, rutin was the most abundant followed by catechin (Table 1 and S1 Table). Kaempferol was not found in these rosehips unlike *Rosa canina* extract from Serbia, studied by Tumbas *et al*. [19] Rutin acts by scavenging free radicals and also by chelating metals [8] and it has been implemented in ROS related diseases, such as gastric lesions and diabetes, based on its scavenging properties on oxidizing species. Rutin also attenuates hypoxic pulmonary and vasoconstriction [37].

**Table 1:** Chemical formula, concentration and total antioxidant capacity of the different extract fractions.

	Antioxidant compound	Concentration^a^	Total antioxidant capacity^b^
Fr 2	Ascorbic acid	101±1	38.5±0.2
Neutral polyphenols Fr 3	Myricetin	5.4±0.2	
	Rutin	22±1	
	Catechin	11.9±0.5	
	Quercetin	1.5±0.2	
	Total	40.8	4.5±0.2
Acidic polyphenols Fr 4	Vanillic acid	260±30	
	Caffeic acid	2±1	
	Syringic acid	110±10	
	Gallic acid	298±2	
	Ellagic acid	80±4	
	Protocatechuic acid	210±20	
	Total	960	3.0±0.1

^a^ Fr 2 and Fr 3 in mg/kg dry fruit; Fr 4 in μg/kg dry fruit.

^b^ mg vit. C/g dry fruit

Demir *et al*. [18] found that catechin concentration presents a great variability in different *Rosa* species from Turkey (1.65 to 39.90 mg/g). Similar to what happened with vitamin C, the reasons for these variations could be the climatic diversity between different geographic areas, environmental factors (i.e. light, temperature, soil nutrients…) and fruit maturity, which can affect phenolic metabolism and conversion. Catechin antioxidant action is very interesting, it is capable of scavenging free radicals by electron transfer processes.

The catechol oxidation mechanism occurs in sequential steps related to catechol and resorcinol groups and the oxidation is pH dependent [38]. The concentrations of myricetin and quercetin were much lower than that of rutin and catechin (Table 1 and S1 Table). Myricetin chemical structure is characteristic, the combined contribution of hydroxyl groups at 3,5 positions and three continuous hydroxyl groups at position 3´,4´ and 5´ can increase its antioxidant efficiency, but the presence of 6 hydroxyl groups can decrease its hydrophobicity which could be a negative factor for the biological action of this polyphenol [39]. Quercetin is the most abundant flavonoid that accumulates in superior plants where it forms glucosides, such as rutin, with great sugars variety [40], however, its concentration is the smallest one found among the neutral phenolic compounds of this rosehip sample. Quercetin and its derivative quercetin-3-O-glucuronide inhibit ROS overproduction, by chemoprotection of mitochondrial function through antioxidative actions. Free fatty acids facilitate ROS formation by suppression of electron transport in respiration chain increasing ROS levels, which induces a potential collapse of mitochondrial membrane [41]. The total antioxidant capacity of this fraction was 4.5±0.2 mg of vitamin C/g of dry sample.

Although a higher number of acidic polyphenols were found, total concentration of this fraction was the smallest one (960 μg/Kg of dry matter). The most abundant compound of this fraction was gallic acid while caffeic acid was present in a really low concentration; coumaric acid was not detected (Table 1 and S1 Table). Total antioxidant capacity of this fraction was 3.0±0.1 mg of vitamin C/g of dry sample. Unlike what happened in neutral polyphenols, little is known about the bioactive *in vivo* forms of acidic phenolic fraction and its contribution to disease prevention. Rechner *et al*. [42] among others, have evidence about the metabolism, in the human body, of acidic phenols, through conjugation reactions altering its initial structure. This increases or decreases the bioactivity of the initial acidic phenols [43].

#### Cytotoxicity and apoptosis studies

**Fig 2:**
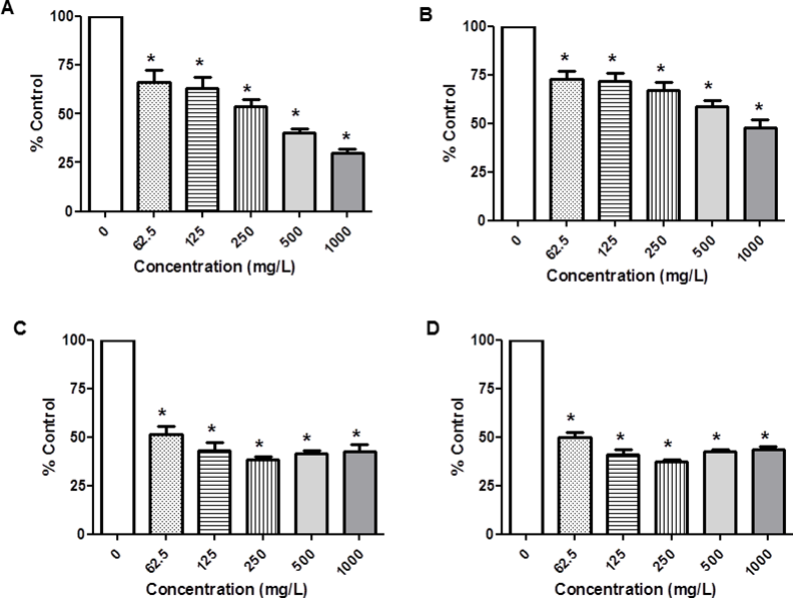
**Caco-2 cells (PD7) cell growth (%)** treated with different concentrations (0–62.5-125-250-500-1000 mg/L) of Fr 1: total extract (A), Fr 2: vit. C (B), Fr 3; neutral polyphenols (C) and Fr 4: acid polyphenols or flavonoids (D) from rosehip (*Rosa canina* L) after 72 h. Values are means ± SEM of three independent experiments, each performed in six repetitions. *p <0.05 compared with control (without treatment).

**Fig 3:**
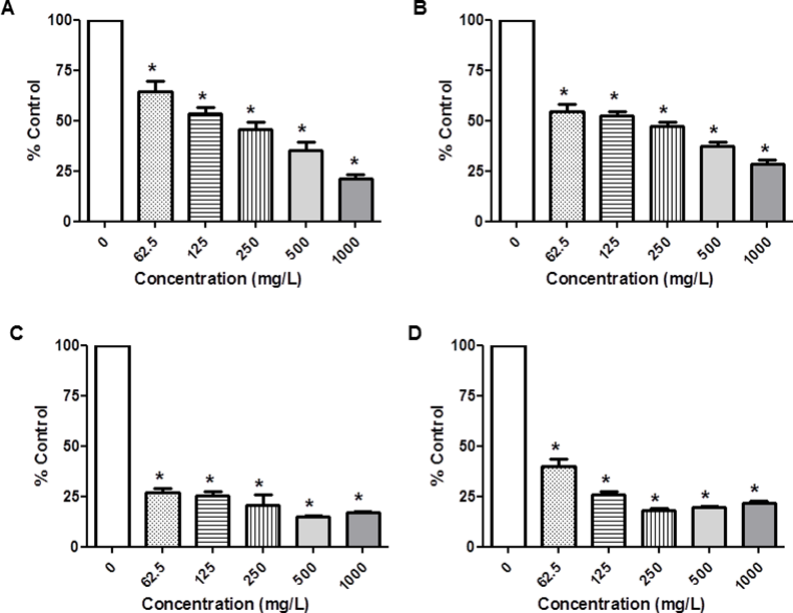
**Caco-2 cells (TC7) cell growth (%)** treated with different concentrations (0–62.5-125-250-500-1000 mg/L) of Fr 1: total extract (A), Fr 2: vit. C (B), Fr 3; neutral polyphenols (C) and Fr 4: acid polyphenols or flavonoids (D) from rosehip (*Rosa canina* L) after 72 h. Values are means ± SEM of three independent experiments, each performed in six repetitions. *p < 0.05 compared with control (without treatment).

The cellular effects of *Rosa canina* fractions have been tested against human colon cancer cell lines (Caco-2). The results are expressed in terms of % cell growth (Figs 2 and 3 and S1 Dataset), which were obtained after exposure to the fractions for 72 h, using the well-established sulforhodamine B assay (see Material and Methods for details). Exposure of Caco-2 cell lines to increasing concentrations (0–1000 mg/L) of all roseship fractions led to a remarkable inhibition of cell growth (Figs 2 and 3 and S1 Dataset).

**Fig 4:**
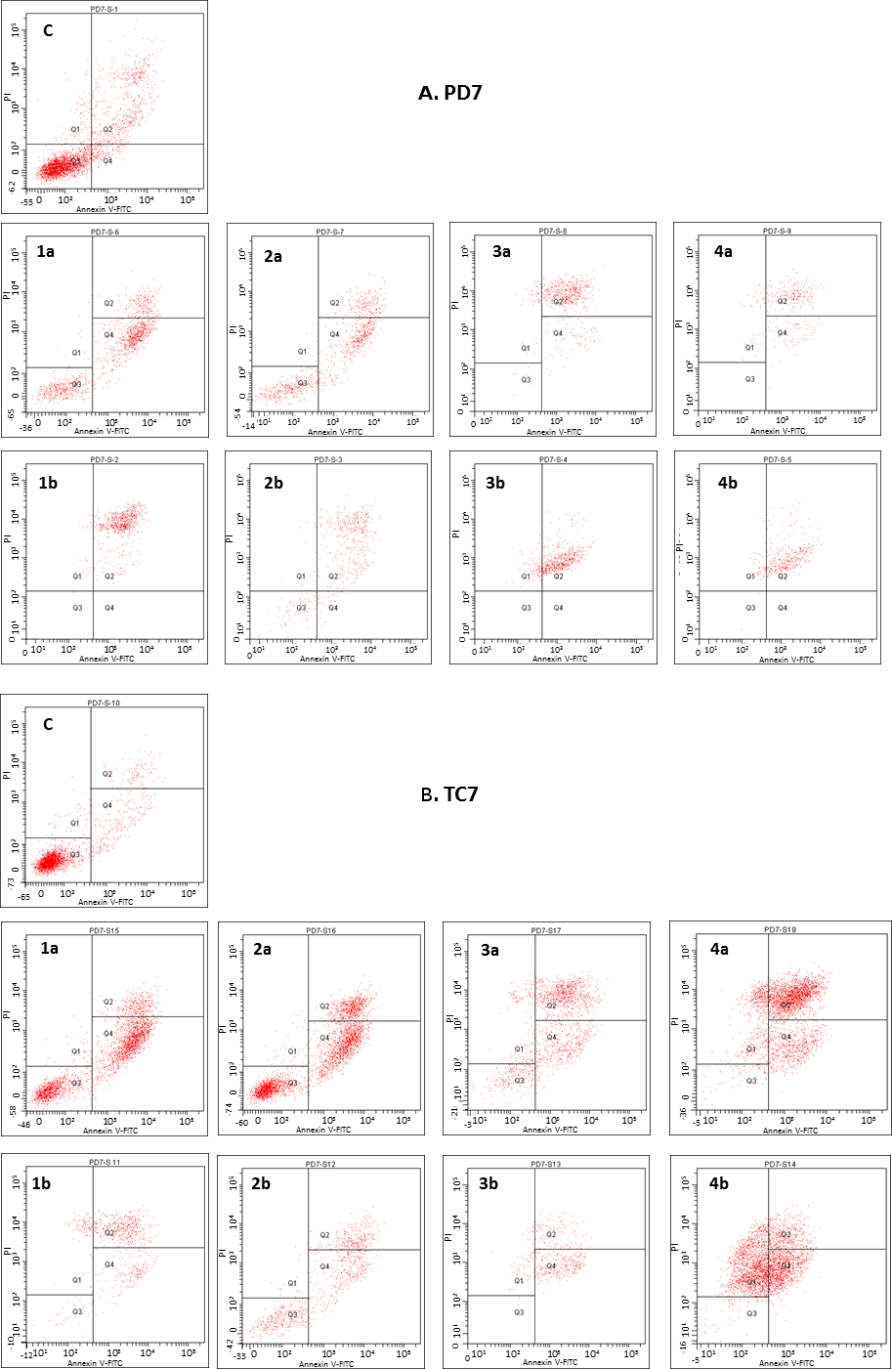
**Quantitative flow cytometry analyses** using propidium (PI) uptake and annexin V staining in PD7 (A) and TC7 (B) colon cancer cells treated with two concentrations (a: 125 and b: 1000 mg/L) of 1 (Fr 1: total extract), 2 (Fr 2: vit. C), 3 (Fr 3: neutral polyphenols) and 4 (Fr 4: acid polyphenols or flavonoids) from rosehip (*Rosa canina* L) after 72 h. In control (C), the cells are without treatment. Experiments were performed in triplicate.

As cancer is characterized by uncontrolled cellular proliferation, there is considerable interest in treatment-induced apoptosis. In light of this, we tested the ability of different fractions of *Rosa canina* to inhibit human tumor cell proliferation by inducing apoptosis using the Annexin-V/propidium iodide double-staining assay, which are well-established biomarkers of cell death [44] over 72 h (Fig 4A and 4B and S2 Dataset). During the early stages of apoptosis, the cell membrane loses asymmetry and the membrane phospholipids phosphatidylserine (PS) is translocated from the cytoplasmic to the extracellular side. Annexin-V (FITC conjugated) specifically binds to PS exposed on the cell surface and can be detected by flow cytometry, thereby allowing the identification of early stage apoptic cells. Viable cells with intact membranes and cells undergoing early apoptosis but with relatively intact membranes exclude propidium iodide, whereas the membranes of dead and damaged cells are permeable to PI and are also stained with annexin-V [44].

In this way, the different plant fractions at concentrations that decreased the cell viability were assayed. Data reported in Table 2 and Fig 4A and 4B (S2 Dataset) show that treatment with the different plant fractions for 72 h produces much higher amounts of apoptosis than in cells without treatment (control) for both cancer cell lines (PD7 and TC7), thus indicating their ability to induce cell death by an apoptotic pathway. At 125 mg/L concentration, fractions 1 and 2 induce a significantly higher degree of early apoptosis compared with control, whereas the treatment of cells with fractions 3 and 4 induces late apoptosis. On the other hand, at 1000 mg/L concentration, fractions 1 and 2 induce late apoptosis (Table 2, Fig 4A and 4B and S2 Dataset). To summarize, the results obtained show that these fractions of *Rosa canina* are able to induce cell death by activating apoptic pathways, thereby reducing their ability to non-selectively react with biological targets to cause necrosis and its related side effects.

**Table 2:** Summary of PD7 and TC7 colon cancer cells treated with two concentrations (a: 125 and b: 1000 mg/L) of rosehip (*Rosa canina* L) fractions after 72 h. In control, the cells are without treatment.

	Fractions	Live (%)	Early apoptic (%)	Late apoptic (%)	Necrotic (%)
PD7	Control	76.4	4.3	16.9	2.4
	Fr 1a	29.1	53.7	16.3	0.9
	Fr 2a	35.1	44.9	18.8	1.2
	Fr 3a	7.7	12.2	79.0	1.0
	Fr 4a	0.0	22.3	64.2	13.5
	Fr 1b	0.6	0.1	96.0	3.3
	Fr 2b	14.4	6.2	76.5	2.9
	Fr 3b	0.3	0.0	84.8	14.9
	Fr 4b	0.5	0.0	87.6	11.9
TC7	Control	88.3	7.1	3.1	1.5
	Fr 1a	35.7	52.3	10.8	1.2
	Fr 2a	40.6	39.5	18.9	1.0
	Fr 3a	26.9	12.2	56.4	4.4
	Fr 4a	28.1	27.0	41.1	3.8
	Fr 1b	5.4	22.4	59.9	12.3
	Fr 2b	32.0	35.6	30.4	1.9
	Fr 3b	14.0	26.4	50.3	9.3
	Fr 4b	1.9	19.5	62.3	16.2

We subsequently analyzed the effects of the plant extracts on cell-cycle progression in both cancer cell lines after 72 h of treatment. Cell-cycle analysis was performed by using flow cytometry to assess the DNA content of cells stained with propidium iodide. The fluorescence intensity of the stained cells at certain wavelengths correlates with the amount of DNA contained therein. As the DNA content of cells doubles during the S phase of the cell cycle, the relative amount of cells in the G_0_ phase and G_1_ phase (before the S phase) can be determined, as the fluorescence of cells in the G_2_/M phase will be twice as high as that of cells in the G_0_/G_1_ phase. Cell cycle anomalies can indicate various kinds of cell damage, for example DNA damage, which cause the interruption of the cell cycle at certain checkpoints to prevent transformation into a cancer cell (carcinogenesis). In this way, studies of Hu *et al*. [45] revealed that anti-cancer agents arrest the cell cycle at the G_0_/G_1_, S or G_2_/M phase and then induce apoptotic cell death. The cell cycle arrest has become an appreciated target for management and treatment of tumor cells with cytotoxic agents [46]. The fluorescence intensity of sub G_0_ cell fraction represents the apoptotic cell population [47].

**Fig 5:**
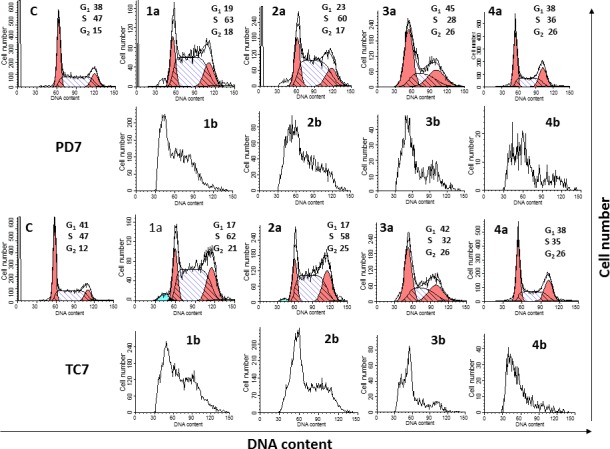
**Cell-cycle analysis after treatment with two concentrations** (a: 125 and b: 1000 mg/L) of Fr 1: total extract (1), Fr 2: vit. C (2), Fr 3: neutral polyphenols (3) and Fr 4: acid polyphenols or flavonoids (4) from rosehip (*Rosa canina* L) after 72 h. In control (C), the cells are without treatment. Cell cycle and DNA fragmentation were determined by propidium iodide staining. Percentages of G_1_, S and G_2_-phase are shown when possible. Experiments were performed in triplicate.

In the present study, Fig 5 (S2 Dataset) shows a graphic of PD7 and TC7 cells treated with the different fractions of *Rosa canina*. Fractions 1 and 2 at 125 mg/L increase the S phase with a decrease in the G_0_/G_1_ involving an S-phase arrest. A decrease in the population in the S-phase, resulting in a concomitant increase in the G_2_ phase is detected in fractions 3 and 4 after incubation with cells that could prevent the entry of cells into the subsequent phase. This fact could be in accordance with the late apoptosis found for these fractions. At 1000 mg/L concentration, all fractions produce important disturbances in the cell cycle that could affect cell cycle progression. This event could be related to the increase in late apoptosis (Table 2 and S1 Dataset).

#### Antioxidant activity of *Rosa canina* fractions

Oxidative stress is induced by reactive oxygen species such as superoxide anions (-OOH), hydrogen peroxide (H_2_O_2_) and hydroxyl radicals (-OH). ROS are generated in aerobic respiration and metabolism [48] and modulated by antioxidant enzymes and non-enzymatic scavengers [49]. Natural antioxidants obtained from plants and vegetables are generally needed to counteract the damage of ROS to cells. The involvement of ROS and free radicals in the pathogenesis of certain human diseases, including cancer, is becoming increasingly recognised [50]. Thus, low-to-moderate levels of ROS are essential for cellular proliferation, differentiation, and survival [51].

**Fig 6:**
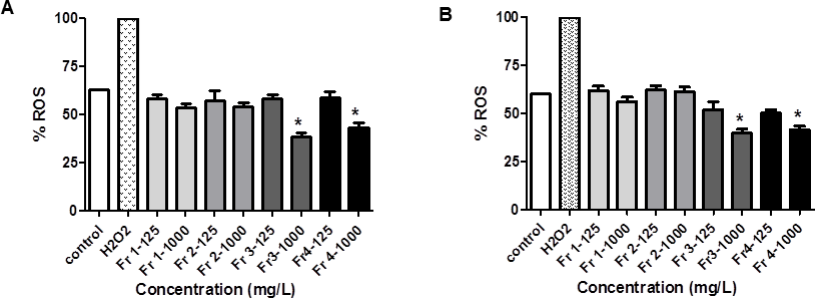
**Effect of plant fractions (1–4) on the generation of ROS in Caco-2 cells:** (A: PD7 and B: TC7) at 125 and 1000 mg/L for 72 h treatment. % ROS production compared to the cells treated for 20 min with 20 mM H_2_O_2_ (positive control, H_2_O_2_). Negative control (without H_2_O_2_, control). Values are means ± SEM of three independent experiments, each performed in six repetitions. *p < 0.05 compared with negative control (without H_2_O_2_).

In order to assess the cytotoxicity mechanism produced by the *Rosa canina* fractions on Caco-2 cells, this study investigated the role of oxidative stress in terms of ROS generation. The production of ROS demonstrated that Caco-2 cells treated with H_2_O_2_ 20 mM and different plant fractions showed a reduction in the production of oxidizing species compared to positive control (with 20 mM H_2_O_2_) (Fig 6A and 6B and S2 Table). The values were similar to the negative control (without H_2_O_2_) being slightly lower at 1000 mg/L concentration to fractions 3 and 4.

## Conclusions

Our results indicated that both vitamin C and polyphenols (neutral and acidic) from rosehips, have antioxidant and antiproliferative effects against cancer cells (Caco-2). These effects are provided by vitamin C, polyphenols and flavonoids both together (fraction 1) and separately (fractions 2–4). These effects improve cancer disease by the apoptotic pathway. Progress in this subject can direct the therapeutic strategies towards consumption of functional foods. In this way, *Rosa canina* is emerging as a food with benefits for nutrition and health. Further studies in this regard are the aim of our group.

## Supporting Information

S1 Dataset Results of the cytotoxicity and apoptosis. Data of all the concentrations of rosehips fractions tested in two human colon cancer cell lines (Caco-2).
(XLS)

S2 Dataset Quantitative flow cytometry and cell cycle analyses. Results of all the concentrations of rosehips fractions tested in two human colon cancer cell lines (Caco-2).
(DOCX)

S1 Table Results of antioxidants in rosehips obtained by HPLC. In this table are all the results corresponding to 4 injections of each sample.
(DOCX)

S2 Table Effects of plant fractions on the generation of ROS in Caco-2 cells. Results of all the concentrations of rosehips fractions tested in two human colon cancer cell lines (Caco-2).
(XLSX)
