# Supplementary material for: Rosa canina Extracts Have Antiproliferative and Antioxidant Effects on Caco-2 Human Colon Cancer
Source: PLoS One. 2016 Jul 28;11(7):e0159136. doi: 10.1371/journal.pone.0159136 (PMC4965184; doi:10.1371/journal.pone.0159136)

**S2 Dataset. Quantitative flow cytometry and cell cycle analyses.** Results of all the concentrations of rosehips fractions tested in two human colon cancer cell lines (Caco-2).

Caco-2 PD7 p.80 Treatment: 72 h **CONTROL**


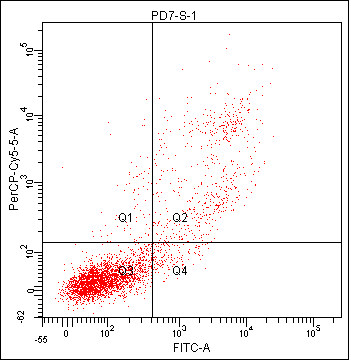

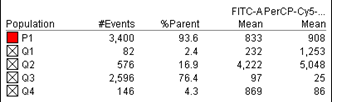


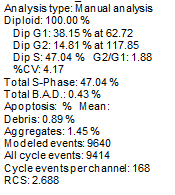


Caco-2 PD7 p.80 Treatment: 72 h **Vit C 125 mg/L (1a)**


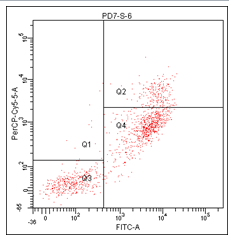

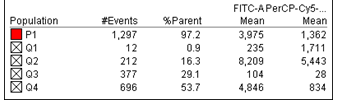


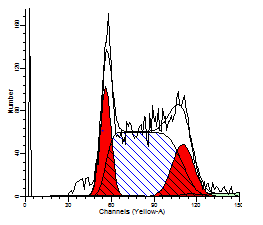

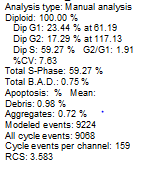


Caco-2 PD7 p.80 Treatment: 72 h **Vit C 125 mg/L (2a)**


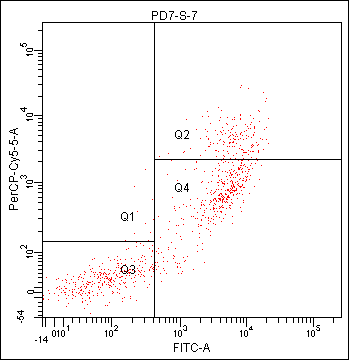

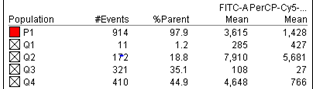


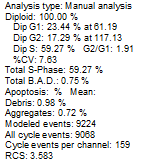


Caco-2 PD7 p.80 Treatment: 72 h **Fracción 3 125 mg/L (3a)**


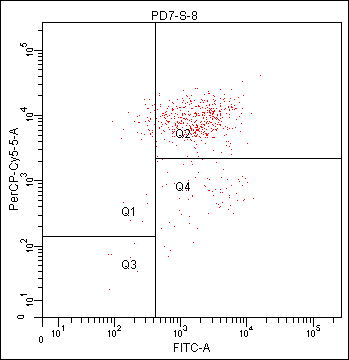

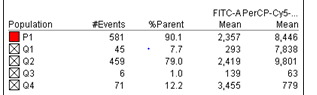


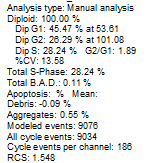


Caco-2 PD7 p.80 Treatment: 72 h **Fracción 4 125 mg/L (4a)**


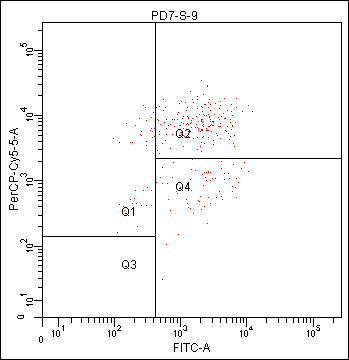

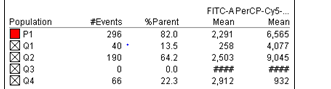


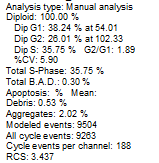


Caco-2 PD7 p.80 Treatment: 72 h **Extracto total 1000 mg/L (1b)**


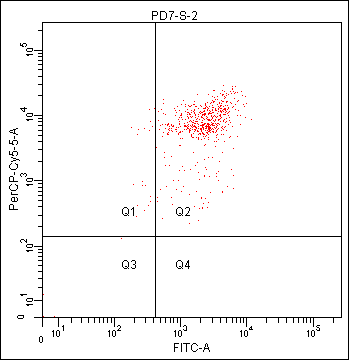

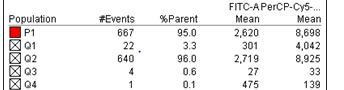

Caco-2 PD7 p.80 Treatment: 72 h **Vit C 1000 mg/L (2b)**


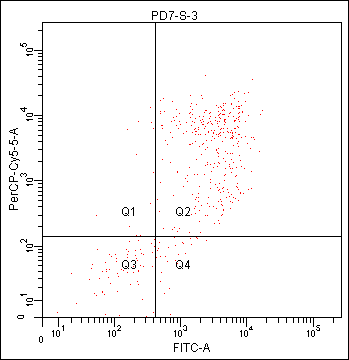

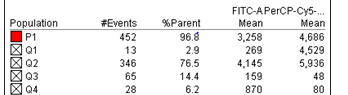

Caco-2 PD7 p.80 Treatment: 72 h **Fracción 3 1000 mg/L (3b)**


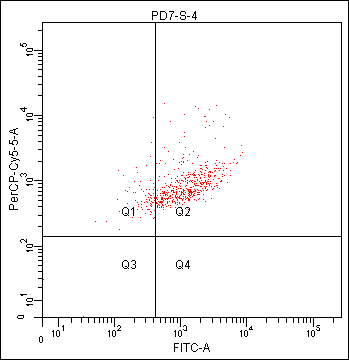

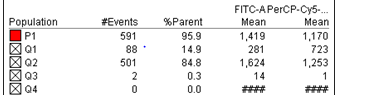

Caco-2 PD7 p.80 Treatment: 72 h **Fracción 4 1000 mg/L (4b)**


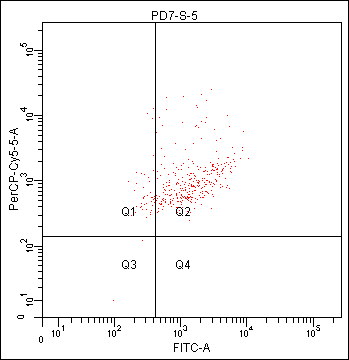

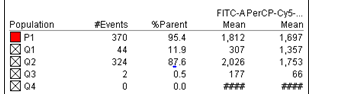

Caco-2 TC7 p.26 Treatment: 72 h **CONTROL**


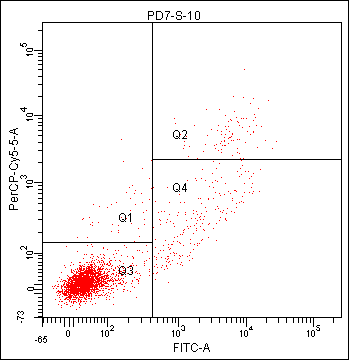

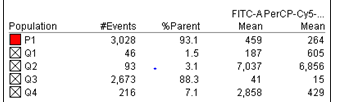


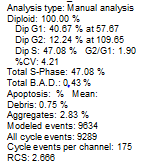


Caco-2 TC7 p.26 Treatment: 72 h **Extracto total 125 mg/L (1a)**


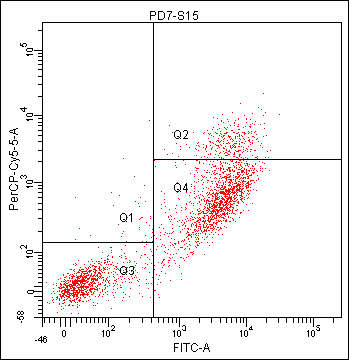

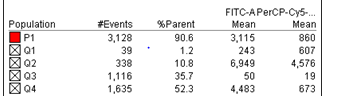


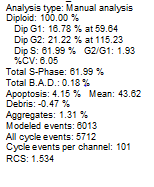


Caco-2 TC7 p.26 Treatment: 72 h **Vit C 125 mg/L (2a)**


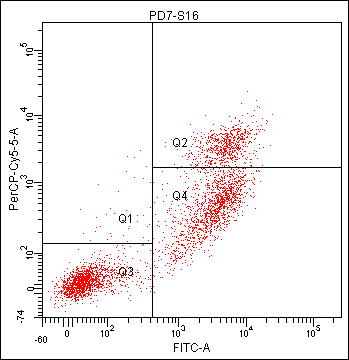

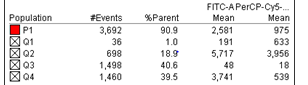


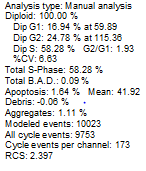


Caco-2 TC7 p.26 Treatment: 72 h **Extracto 3 125 mg/L (3a)**


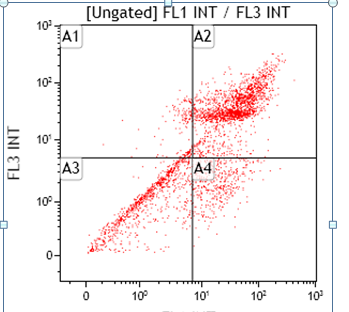

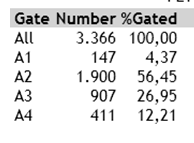


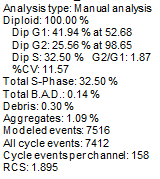


Caco-2 TC7 p.26 Treatment: 72 h **Extracto 3 125 mg/L (4a)**


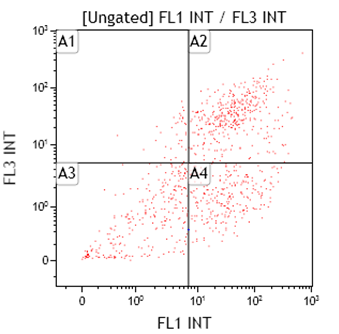

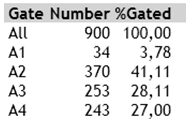


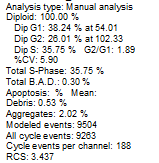


Caco-2 TC7 p.26 Treatment: 72 h  **Extracto total 1000 mg/L (1b)**


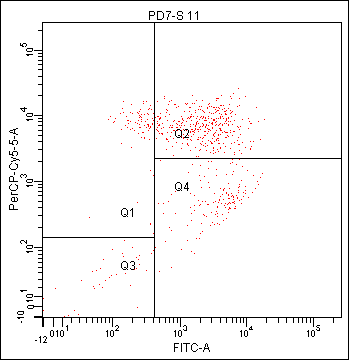

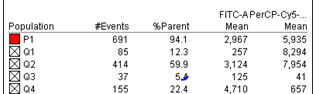

Caco-2 TC7 p.26 Treatment: 72 h **Vit C 1000 mg/L (2b)**


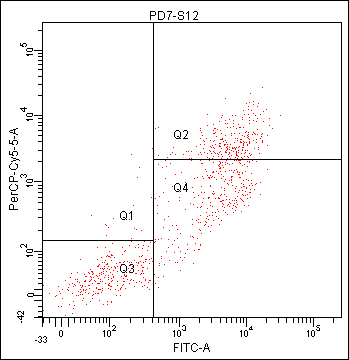

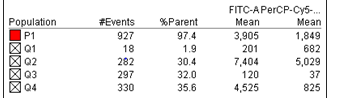

Caco-2 TC7 p.26 Treatment: 72 h **Extracto 3 1000 mg/L (3b)**


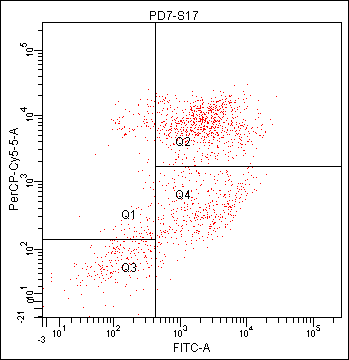

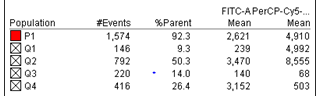

Caco-2 TC7 p.26 Treatment: 72 h **Extracto 4 1000 mg/L (4b)**


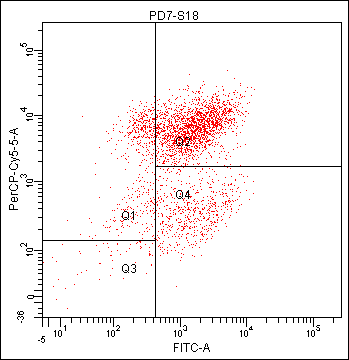

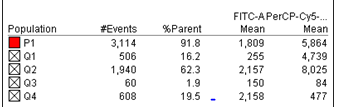

Supplement: S2 Dataset — Results of all the concentrations of rosehips fractions tested in two human colon cancer cell lines (Caco-2). (DOCX) [file pone.0159136.s002.docx]
