# Supplementary material for: Rosa canina Extracts Have Antiproliferative and Antioxidant Effects on Caco-2 Human Colon Cancer
Source: PLoS One. 2016 Jul 28;11(7):e0159136. doi: 10.1371/journal.pone.0159136 (PMC4965184; doi:10.1371/journal.pone.0159136)
Supplement: S1 Table — In this table are all the results corresponding to 4 injections of each sample. (DOCX) [file pone.0159136.s003.docx]

**S1 Table. Results of antioxidants in rosehips obtained by HPLC**. In this table are all the results corresponding to 4 injections of each sample.

| **mg antioxidant/ Kg dry fruit** | | | | | |
| --- | --- | --- | --- | --- | --- |
|  | **Injection 1** | **Injection 2** | **Injection 3** | **Injection 4** | **Media** |
| Myrcetin | 5,619799917 | 5,171665771 | 5,343745709 | 5,288004548 | 5,272855144 |
| Rutin | 24,29299159 | 21,2884697 | 22,03066983 | 21,58441574 | 22,29913672 |
| Catechin | 12,69735498 | 11,68532614 | 11,72966358 | 11,66377435 | 11,94402976 |
| Quercetin | 1,300899454 | 1,76242379 | 1,422272258 | 1,558327473 | 1,510980744 |
| Vanillic acid | 0,23938574 | 0,289355177 | 0,224939854 | 0,288011624 | 0,260423099 |
| Caffeic acid | 0,001579512 | 0,001964759 | 0,001246799 | 0,003243078 | 0,002008537 |
| Syringic acid | 0,101819797 | 0,112511922 | 0,117125816 | 0,10445397 | 0,108977876 |
| Gallic acid | 0,298447932 | 0,300233867 | 0,298642055 | 0,296157275 | 0,298370282 |
| Ellagic acid | 0,074781566 | 0,07623637 | 0,080040173 | 0,084302657 | 0,078840192 |
| Protocatechuic acid | 0,226590414 | 0,213391283 | 0,200596967 | 0,186023592 | 0,206650564 |
| Ascorbic acid | 100,7233514 | 99,84243452 | 103,2673926 | 101,0638871 | 101,2242664 |
